# Three-Dimensional Arterial Pulse Signal Acquisition in Time Domain Using Flexible Pressure-Sensor Dense Arrays

**DOI:** 10.3390/mi12050569

**Published:** 2021-05-17

**Authors:** Jianzhong Chen, Ke Sun, Rong Zheng, Yi Sun, Heng Yang, Yifei Zhong, Xinxin Li

**Affiliations:** 1 State Key Laboratory of Transducer Technology, Shanghai Institute of Microsystem and Information Technology, Chinese Academy of Sciences, Shanghai 200050, China; chenjzh@mail.sim.ac.cn (J.C.); sunke@mail.sim.ac.cn (K.S.); sunyi@mail.sim.ac.cn (Y.S.); h.yang@mail.sim.ac.cn (H.Y.); 2 School of Information Science and Technology, ShanghaiTech University, Shanghai 201210, China; 3 School of Microelectronics, University of Chinese Academy of Sciences, Beijing 100049, China; 4 Department of Nephrology, Longhua Hospital, Shanghai University of Traditional Chinese Medicine, Shanghai 200032, China; zrong_md@shutcm.edu.cn

**Keywords:** pulse diagnosis, traditional Chinese medicine, MEMS pressure sensor, dynamic pulse width, pulse wave velocity

## Abstract

In this study, we developed a radial artery pulse acquisition system based on finger-worn dense pressure sensor arrays to enable three-dimensional pulse signals acquisition. The finger-worn dense pressure-sensor arrays were fabricated by packaging 18 ultra-small MEMS pressure sensors (0.4 mm × 0.4 mm × 0.2 mm each) with a pitch of 0.65 mm on flexible printed circuit boards. Pulse signals are measured and recorded simultaneously when traditional Chinese medicine practitioners wear the arrays on the fingers while palpating the radial pulse. Given that the pitches are much smaller than the diameter of the human radial artery, three-dimensional pulse envelope images can be measured with the system, as can the width and the dynamic width of the pulse signals. Furthermore, the array has an effective span of 11.6 mm—3–5 times the diameter of the radial artery—which enables easy and accurate positioning of the sensor array on the radial artery. This study also outlines proposed methods for measuring the pulse width and dynamic pulse width. The dynamic pulse widths of three volunteers were measured, and the dynamic pulse width measurements were consistent with those obtained by color Doppler ultrasound. The pulse wave velocity can also be measured with the system by measuring the pulse transit time between the pulse signals at the brachial and radial arteries using the finger-worn sensor arrays.

## 1. Introduction

The wrist pulse waveform is an important physiological signal generated by the periodic contraction and dilation of arteries and contains abundant information regarding an individual’s physical condition. Specifically, many characteristics of the pulse waveform can be assessed to diagnose health conditions, such as hypertension [1], arterial stiffness [2], vascular aging [3], and atrial fibrillation [4], as well as other cardiovascular health information, such as cardiac output [5]. Pulse signal analysis is already widely applied for arterial stiffness assessment in assorted commercial instruments, such as the Complior and SphygmoCor [6]. Furthermore, many consumer electronics and wearable devices, such as smart wristbands and watches, estimate heart rate and other cardiovascular information from pulse waves [7].

In a similar vein, pulse diagnosis plays a dominant role in traditional Chinese medicine (TCM). Traditional Chinese doctors apply pressure at one of three levels—superficial pressure, medium pressure, or deep pressure—to the radial artery at various points (inch, bar, cubit) and use the feel of the resulting pulse waveform to assess the health of patients [8]. However, pulse diagnosis in TCM requires significant skill, and no objective data can be obtained. Thus, there is an urgent need for an instrument that can effectively assist in pulse diagnosis by recording and digitally analyzing the pulse signal.

Many sensor types have been tested for pulse-taking at the radial artery, including optical [9], polyvinylidene fluoride (PVDF) [10,11], ultrasonic Doppler [12,13], piezoelectric [14,15,16], strain gauges [17], and pressure sensors [18]. Recently, many researchers have examined on binocular vision theory [19], CCD sensors [20], CMOS image sensors [21], 3D digital image correlation methods [22], optical fiber sensors [23,24], millimeter wave devices [25], and RF sensors [26], among other approaches. However, these strategies often ignore static contact pressure, which is important in traditional Chinese pulse diagnosis (TCPD).

With the development of flexible materials, some flexible sensors and sensor arrays have been fabricated for arterial pulse acquisition [17,27,28,29,30,31]. The flexible sensors are thin, soft and stretchable, and show great potential in future wearable devices. However, the consistency, repeatability, and linearity of the flexible sensors are still much lower than silicon MEMS piezoresistive sensors like the ones used in this work. In this paper, we focus our work on making very small silicon MEMS piezoresistive sensors (sensor chip size is only 0.4 mm) that not only simplify the design of circuit system and calibration, but also achieve a dense sensor array to cover a relatively much longer line segment. In the work of [17], the authors used a finger-tip worn flexible strain gauge sensor as a measurement tool for continuous motion activity and health monitoring. It can also be used to measure the strain of skins caused by pulse signals. This work still uses the finger-worn measurement method, but with the single sensor replaced by a dense sensor array for measuring the pulse envelope information.

Despite these varieties of approaches that have been explored, the pulse waveform at the wrist is generally treated as a pressure signal. Accordingly, many pulse acquisition systems relying on pressure sensors have been developed for computerized pulse diagnosis in recent years. These systems have been developed with either a single-point sensor [32,33,34] or three sensors for three locations [35,36]. However, due to the large size of the sensors, these systems can be challenging to position quickly. Furthermore, other information, such as pulse width, often cannot be obtained with pressure sensors alone.

In recent years, some systems have begun to use sensor arrays for pulse measurements [18,37,38,39,40,41], and some of these systems have combined pressure sensors with other sensor arrays to obtain additional information regarding pulse characteristics. However, because the size of the sensor is still relatively large and most sensor arrays are not flexible, the problem of accurately measuring pulse width remains unsolved. Chung-Shing Hu et al., used a 3 × 4 pressure-sensor array with 2.5 mm × 2.5 mm sensor units to determine the temporal and spatial properties of the arterial pulse; the single sensor size was similar to the diameter of the radial artery [18]. Peng Wang et al. proposed a compound pressure signal acquisition system using a sensor array combined with the main sensor and further sub-sensor arrays to solve the problems of sensor positioning and pressure adjustment. However, in their system, the sub-sensor array is not in direct contact with the skin, and the strain gauge sensor array is inflexible [37]. Wang et al. integrated a pressure sensor with photoelectric sensors to create a fusion pulse sensor and designed a multichannel sensor array structure to acquire more pulse information and confirm the pulse position [38]. Liu et al. developed a flexible pressure-sensor array with five sensors for accessing depth information. The compound pressure sensor consisted of a piezoelectric sensor and a piezoresistive sensor, which were used independently to measure the dynamic pulse wave and the static pressure [39]. Chen et al. proposed a system using a micro-electro-mechanical system (MEMS) pressure-sensor array and measured 3D wrist pulse waves to assess pulse width changes before and after exercise. However, the single sensor size is 5.5 mm × 3.6 mm—nearly twice the diameter of the radial artery—which compromises the accuracy of the measurement [40].

Most existing wrist pulse signal acquisition devices and systems suffer from several limitations. First, the sensor sizes in conventional pulse acquisition systems are often much larger than the diameter of the radial artery (approximately 2–3 mm), which makes it difficult to achieve accurate measurements. Second, most pulse diagnosis instruments use a single pulse sensor for single-point measurement; only a few pulse diagnosis instruments can perform three simultaneous measurements, which are needed to assess the pulse at the inch, bar, and cubit. Finally, most current pulse diagnosis instruments can obtain the pulse waveform, pulse rate, and pulse rhythm, but few can accurately and reliably quantify pulse width, which is an important part of TCPD and can help distinguish between flood pulses and thin pulses.

**Figure 1:**
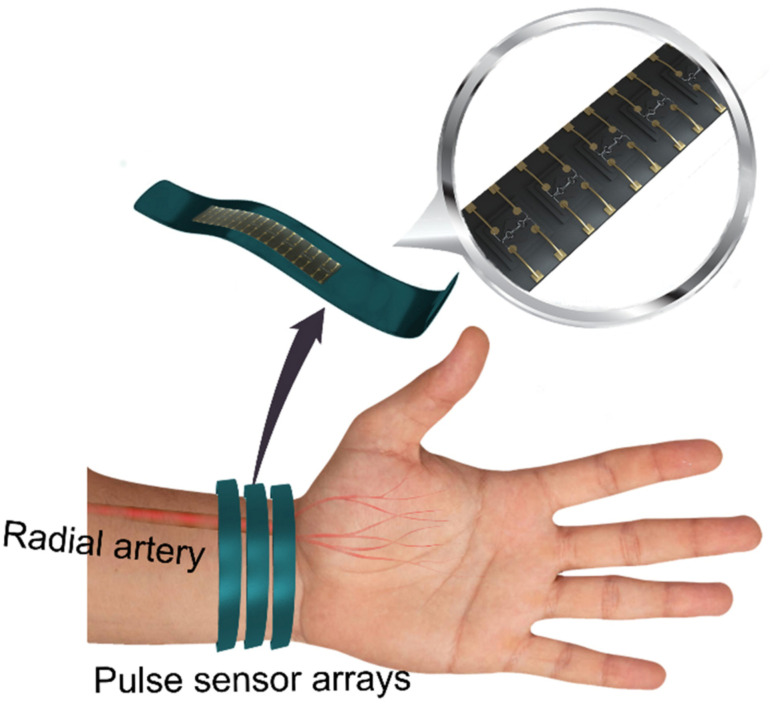
Flexible dense pressure-sensor arrays for three-dimensional arterial pulse signal acquisition.

In this study, flexible dense pressure-sensor arrays were developed by packaging 18 ultra-small MEMS silicon pressure sensors on a flexible printed circuit board (FPC) with a 0.65 mm pitch. Three sensor arrays were used to acquire pulse signals at each relevant site on the radial artery (Figure 1). The sensor arrays were designed to be thin and flexible in order to allow TCM doctors to wear the sensor array on their fingers while palpating the pulses and to facilitate simultaneous pulse signal acquisition. As the sensor array is approximately 2–3 times longer than the pulse signal widths and the pitch is as small as 0.65 mm, the radial pulse sensors can be positioned easily, and the widths of the signals can be effectively distinguished. The strips of sensor arrays are employed in the system to make sure that the pulse signals at inch, bar, cubit can be acquired separately. The three-dimensional pulse envelope images can also be acquired by the system, which show the expansion/contraction of pulse waves in the arteries during the entire pulse cycle and the propagation of pulse signals in the blood vessels. Finally, the time domain parameters of pulse signals, including the radial artery augmentation index (AIr) and pulse wave velocity (PWV), can also be acquired.

## 2. Design and Fabrication of Arterial Pulse Acquisition System

### 2.1. Pulse Sensors Array

**Figure 2:**
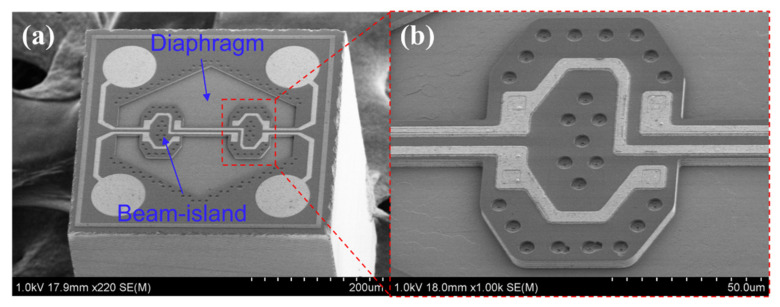
MEMS pressure sensor (0.4 mm × 0.4 mm). (**a**) Scanning electron microscopy image of the MEMS pressure sensor. (**b**) Close-up view of the beam-island structure. (Reproduced with permission from Ref. [43] provided by IOP Publishing, Ltd, United Kingdom of Great Britainand Northern Ireland).

The human radial artery is approximately 2–3 mm in diameter [42]. Thus, a very dense array of pressure sensors is required to measure the pulse width. To achieve high-resolution pulse signal measurement, we used ultra-small absolute pressure sensors for the pulse sensor array, which were previously developed by our research group [43]. Herein, the sensor is absolute pressure sensor, which consists of a very thin but uniform polysilicon diaphragm beneath a bulk-silicon beam-island structure. The thin diaphragm exhibits high sensitivity to pressure, and the beam-island-reinforced structure reduces deflection and improves linearity. The four piezoresistors are integrated to form a fully sensitive Wheatstone bridge in the single-crystalline silicon beam. With the very small chip size of 0.4 mm × 0.4 mm, the MEMS sensors are fabricated using a novel microhole inter-etch and sealing (MIS) process and exhibit high full-scale (FS = 150 kPa) output sensitivity of 108 mV/3.3 V, low hysteresis 0.15% FS, low repeatability error of 0.04% FS, and low nonlinearity of about ±0.1% FS. Figure 2a shows a scanning electron microscope image of the 0.4 mm × 0.4 mm pressure sensor and the beam-island-reinforced structure, as shown in Figure 2b.

**Figure 3:**
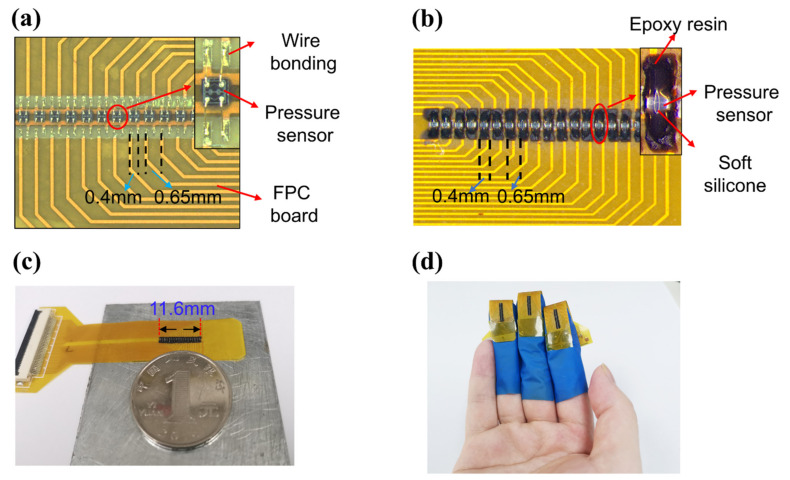
Sensor array packaging procedure. (**a**) Sensors were attached to the FPC board and bonding wire. (**b**) The pressure sensor was coated with silicone and epoxy resin. (**c**) The whole pulse sensor array and FPC board with a coin for scale. (**d**) Pulse sensor arrays are small enough to be worn on the fingers.

The ultra-small pressure sensors were packaged on an FPC to form a dense pressure sensor array. As shown in Figure 3a, 18 MEMS pressure sensors were attached to the FPC substrate and electrically connected by wire bonding. The sensor positions were marked in the FPC substrate to ensure that the sensors are assembled in line and the pitch is 0.65 mm. The bonding wires were then coated with epoxy resin, and the diaphragms of the pressure sensors were coated with soft silicone for force transmission, as shown in Figure 3b. The solidified epoxy resin is stiff. Therefore, the epoxy resin between two sensors was removed to ensure the flexibility of the sensor array. Because the bonding wires are protected by the stiff epoxy resin, when the sensor is stressed, the bonding wire does not move, and the reliability is guaranteed. As shown in Figure 3c, the length of the sensor array was 11.6 mm, which is much larger than the radial artery diameter of 2–3 mm. The sensor arrays pasted on the nitrile gloves can be worn on the fingers, as shown in Figure 3d, which enables simultaneous signal detection during palpation by the TCM practitioner.

The sensor arrays show quite good reliability. All the data shown in this paper were measured with the same sensor array. One bonding wire of the sensor array was broken after the measurement of 330 sets of data. The main failure mechanism of the sensor arrays is the broken of the bonding wires, though the wires were protected with epoxy. The failure issue may be improved by employing the sensors with through silicon via (TSV) vertical interconnection.

**Figure 4:**
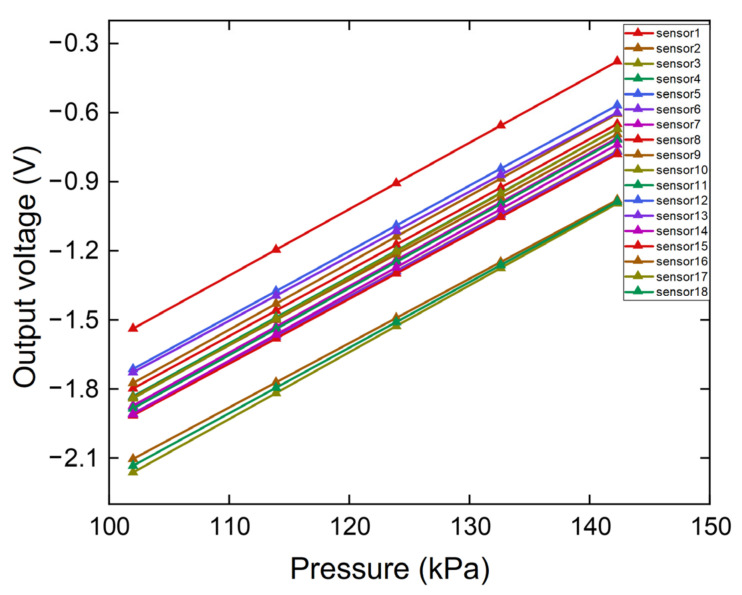
Sensor array output voltage and linear fitting curve.

The packaged pressure sensors were calibrated in a pressure chamber, and the pressure was measured using a digital pressure gauge (Druck DPI-104, General Electric Company, Boston, MA, USA). The pressure sensors are powered by a 3.3 V DC power supply (E3631A, Keysight Technologies, Santa Rosa, CA, USA). The results of the sensitivity test for the array are presented in Figure 4. The average sensitivity of the 18 sensors was 28.62 mV/kPa after the amplifier circuits with 40 times gain. The calibration results were used for pre-processing of the data in subsequent experiments.

### 2.2. Pulse Acquisition System

**Figure 5:**
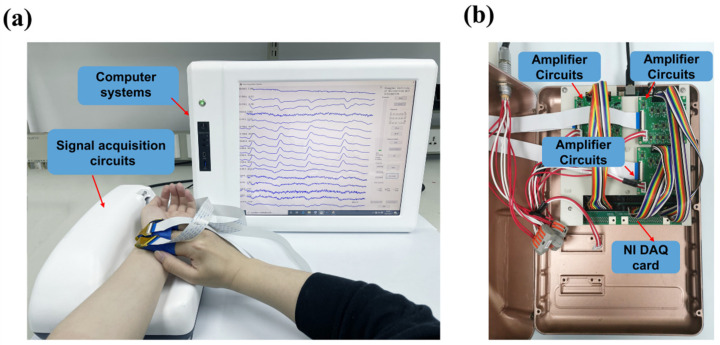
(**a**) The pulse acquisition system. (**b**) Close-up view of the signal acquisition circuits.

The pulse acquisition system was designed to measure multiple parameters of arterial pulse signals, including the pulse width, dynamic width, and PWV. As the PWV is usually faster than 5 m/s, a bandwidth of more than 10 kHz is required for accurate measurement. Thus, each sensor was connected to an AD8221 instrumentation amplifier, which is suitable for Wheatstone bridge sensor applications. The output signals of the pulse sensors were then sampled by a NI6255 data acquisition card (National Instruments Co., Austin, TX, USA), which is capable of processing 80 analog inputs (AI), 24 digital input/outputs (DIO), and a 1.25 MS/s maximum sample rate. In our system, 54 analog inputs were used for the three pulse sensor arrays. Figure 5 shows a picture of the pulse acquisition system, which includes the 54 AD8221 instrumentation amplifiers (Analog Devices, Inc, Norwood, MA, USA), one NI6255 card, and a computer for data processing.

## 3. Results of Three-Dimensional Arterial Pulse Acquisition Studies

### 3.1. Subjects

The arterial pulse acquisition system was tested at the Department of Nephrology, Longhua Hospital, Shanghai. The study was approved by the ethical committee of Longhua Hospital, Shanghai University of Traditional Chinese Medicine. A total of 48 subjects were tested using the system.

### 3.2. Sensor Positioning

**Figure 6:**
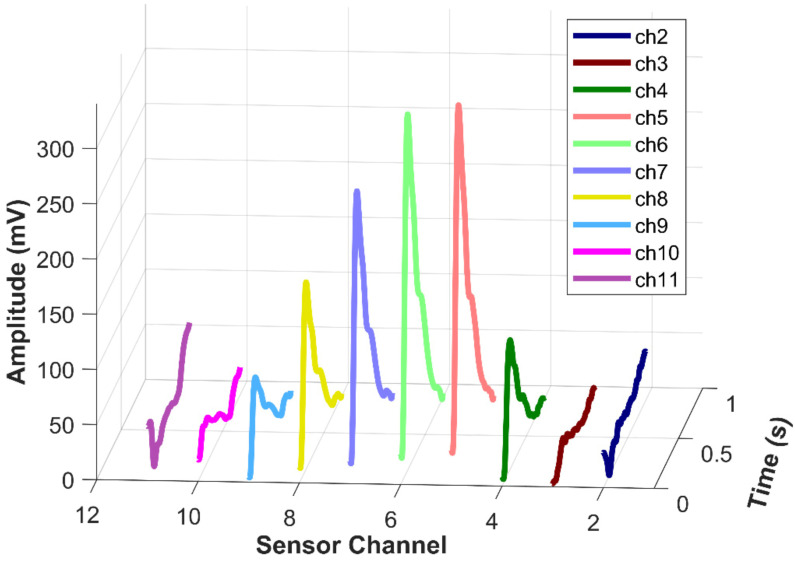
Selected channels of the pulse signal from a subject.

Many existing pulse-test systems have issues with sensor positioning. If the size of the sensor is larger than the diameter of the radial artery, positioning the center of the sensor on the radial artery can be difficult and may prevent accurate signal acquisition. Even if a single small sensor is employed, stable positioning is challenging for thin arteries. To overcome this challenge, our system features 18 ultra-small sensors in an array. The array has an effective span of 11.6 mm, which is 3–5 times the diameter of the radial artery and enables easy pulse detection and precise sensor positioning. Figure 6 shows the pulse signals of a subject. It can be found from the figure that the valid channels can be distinguished easily, which are Channels 4–9 in this case.

### 3.3. Time Domain Waveform Parameters Extraction

**Figure 7:**
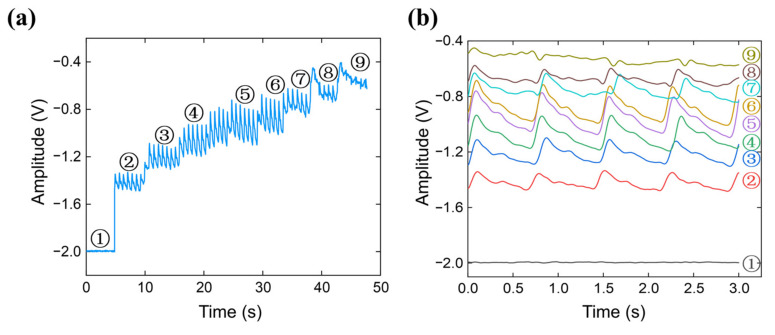
Pulse waves under increasing applied pressures. (**a**) The collected pulse signals. (**b**) The detailed pulse waveforms.

The small size of the sensor array allows TCM doctors to wear the pressure-sensor arrays to acquire pulse signals. Figure 7 illustrates an example of a pulse signal acquired by the finger-worn sensor arrays. In order to determine the arterial pulse waveform at the most suitable pressure, we constantly increase the pressure with our fingers and observe the pulse waveform measured in real-time. As shown in Figure 7a, we applied 9 different pressures to detect pulse waves. As shown in the figure, high-quality pulse signals can be acquired under different applied pressures by well-trained doctors. When the applied pressure is increased, the amplitudes of the pulse signals first increase and reach the maximum, at which the transmural pressure is zero. After that, the amplitudes decrease to zero, because the artery becomes occluded by the external pressure. Figure 7b shows a short interval of the signals at different pressures.

**Figure 8:**
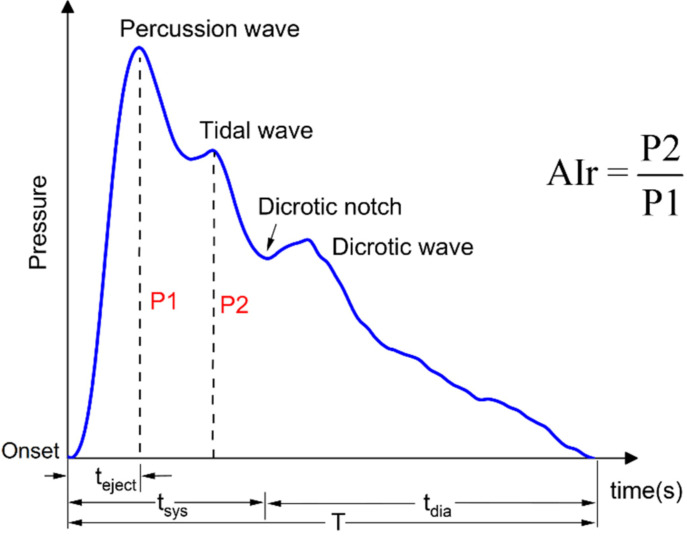
A typical pulse wave.

The maximum waveforms were chosen to represent the typical waveforms and analyzed in the time domain. A typical pulse waveform is shown in Figure 8, which consists of a percussion wave (P1), tidal wave (P2), dicrotic notch, and a dicrotic wave. P1 is caused by an early systolic spike in blood ejection from the left ventricle. P2 is caused by the propagation of blood from the central aorta to the periphery and the reflection of waves from the upper limbs. Multiple parameters can be extracted from the time domain waveform. We measured the radial artery augmentation index (AIr) as a representative example. AIr is defined as the ratio between P2 and P1 [44] and can be used to evaluate arterial stiffness and age-related changes in the artery.

**Figure 9:**
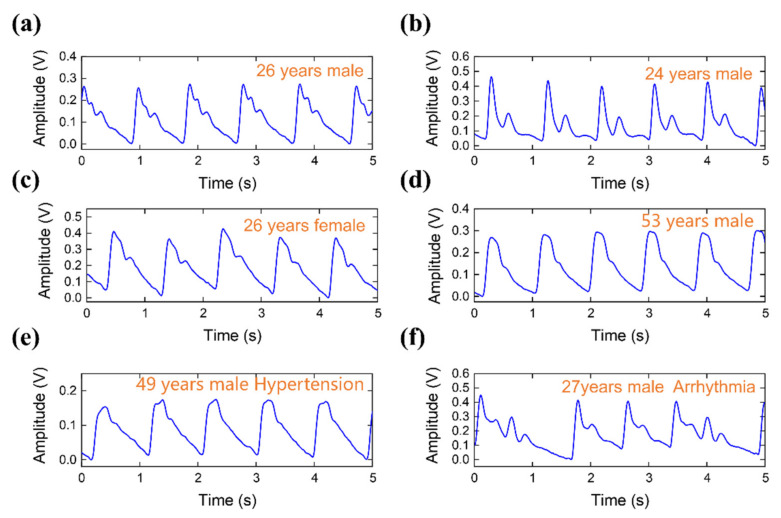
Various pulse waves collected from test subjects with different conditions. Pulse waveforms of (**a**) 26 years male, (**b**) 24 years male, (**c**) 26 years female, (**d**) 53 years male, (**e**) 49 years male with hypertension, (**f**) 27 years male with arrhythmia, respectively.

Figure 9 shows several typical pulse waveforms collected by the pulse acquisition system. Figure 9a was obtained from a healthy 26-year-old man. The percussion wave and dicrotic notch are obvious, which is the typical waveform of a healthy young person. Figure 9b comes from a healthy 24-year-old man. The pulse wave shows a typical double-peak waveform and a low-resistance pulse wave, indicative of good elasticity in the blood vessels and smooth inner blood vessel walls. Figure 9c shows the waveform of a 26-year-old female. And Figure 9d shows the pulse wave measured from a 53-year-old man. Compared with the waveforms in Figure 9a,b, the dicrotic notch is flat and unclear. The changing trend is consistent with the change in the radial artery waveform with increasing age described in the literature [45]. Figure 9e is from a 49-year-old hypertensive male and shows a clear, high-resistance pulse waveform and a flat dicrotic notch. When the elastic modulus of the blood vessel increases, the front of the pulse wave becomes steep, the tidal wave merges into the percussion wave, and the percussion pulse wave widens. Finally, Figure 9f shows an arrhythmia pulse waveform from a 27-year-old volunteer.

**Figure 10:**
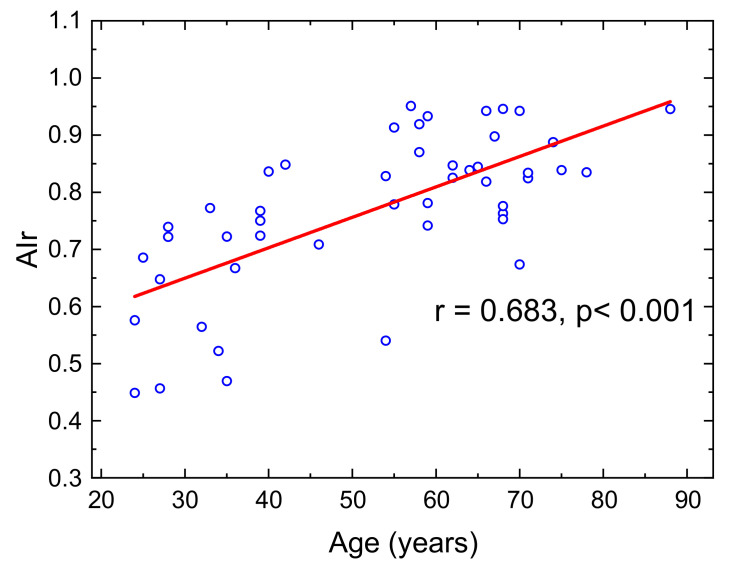
AIr with respect to ages of 48 subjects.

A total of 48 subjects participated in the measurement of AIr. The mean age was 47.0 ± 15.4 years (range 24–88 years). The AIr results for the 48 subjects are shown in Figure 10. There was a moderate correlation between AIr and age across all subjects (r = 0.683, *p* < 0.001), which was comparable to the correlations observed in a previous study [44].

### 3.4. 3D Pulse Envelope Image

**Figure 11:**
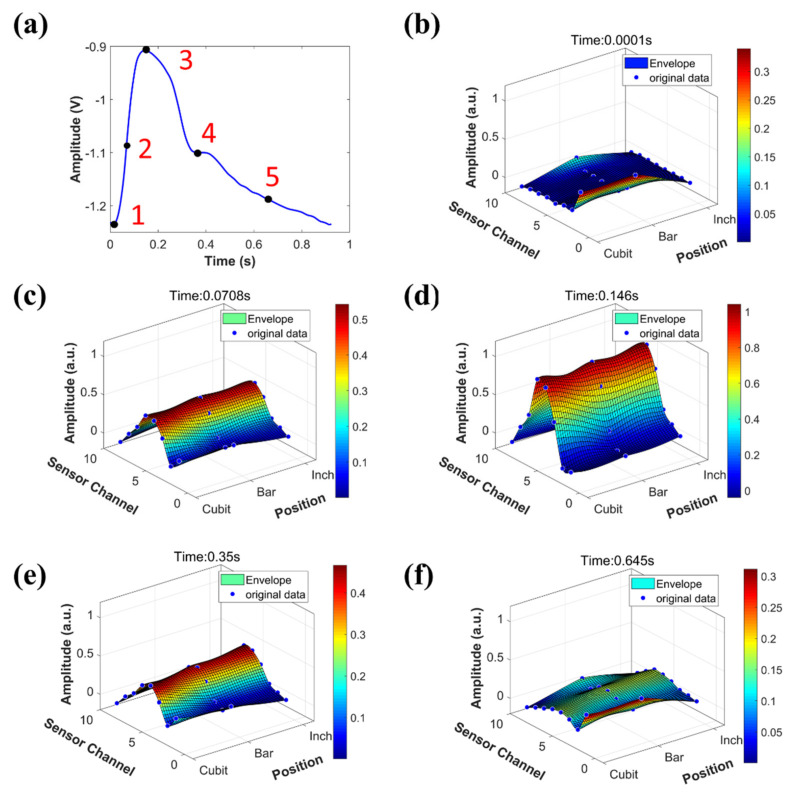
3D pulse envelope images at different timestamps. (**a**) One cycle pulse waveform was collected from the cubit position. (**b**–**f**) are the 3D pulse envelope images at the corresponding time points of 1, 2, 3, 4, 5 in the curve of (**a**), respectively.

The three-dimensional pulse envelope images can be acquired by the system, which shows the expansion and contraction of pulse waves in the arteries during the entire pulse cycle, and the propagation of pulse signals in the blood vessels. The 3D pulse envelope images of a volunteer are shown in Figure 11. Figure 11a shows the corresponding pulse waveform collected from the cubit position. Figure 11b–f are 3D pulse envelope images at five different times marked in Figure 11a, which are onset (Figure 11b), 0.5 times of maximum peak point (Figure 11c), percussion peak point (Figure 11d), dicrotic notch point (Figure 11e), and a point at late diastolic period (Figure 11f). The x-axis shows the positions of the three sensor arrays at the inch, bar, and cubit. The y-axis is the sensor channels. Eight channels are shown in Figure 11, of which about six channels contain valid pulse data. The z-axis shows the normalized pulse signals amplitudes within the range of 0–1 according to the maximum amplitude at each inch, bar, and cubit position. The curve is fitted by the “thinplateinterp” method in Matlab 2019a software (The MathWorks, Inc., Natick, MA, USA). The GIF version of Figure 11 is provided in the Supplementary Materials Figure S1 of the paper, and Figure S2 is the normalized single-point pulse wave of Figure 11a.

### 3.5. Pulse Width and Dynamic Pulse Width

In TCM, both the pulse width and the dynamic pulse width are important parameters for classifying pulse signals. The dynamic pulse width refers to the difference between the maximum and minimum pulse widths during a pulse period. During the systolic period, blood vessels expand to the maximum width because of the ejection of blood from the heart; during the diastolic period, blood vessels gradually return to their minimum diameter. The dynamic pulse width may be a good predictor of arterial stiffness, although further studies are required. The diameter of the radial artery is known to be approximately 2–3 mm with an average change of 0.3–0.4 mm within a pulse cycle [42]. The small pitch of our sensor arrays allows the pulse width and dynamic pulse width to be measured qualitatively.

**Figure 12:**
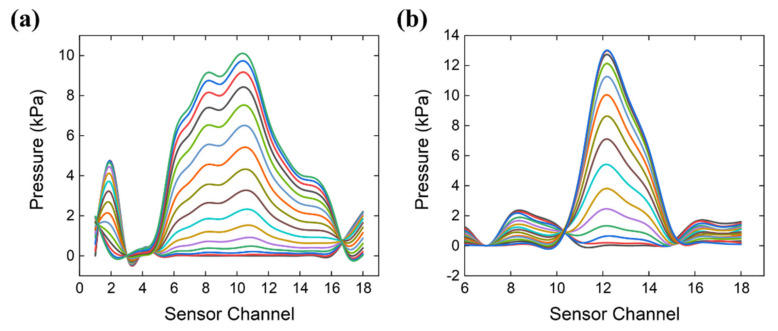
Cross-sectional curve of arterial pulse at different timestamps. Each colored line represents one cross-sectional amplitude curve sampled every six time points during the pulse onset to the percussion peak. The Cross-sectional curve from a subject with (**a**) eight valid sensor channels and (**b**) three valid sensor channels.

**Figure 13:**
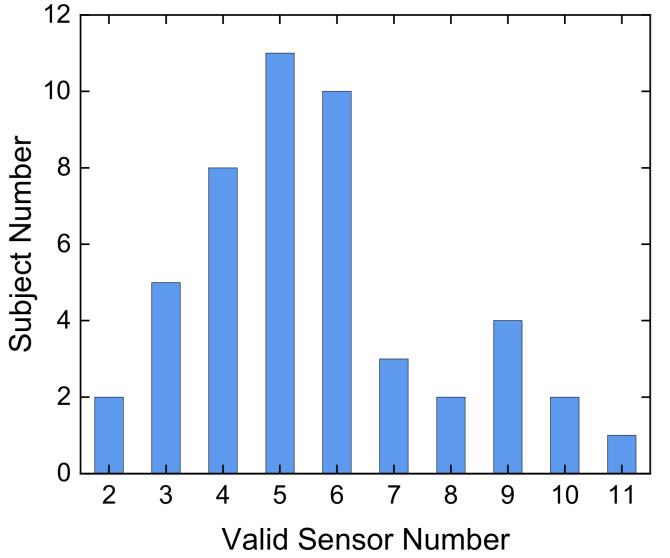
Summary of the pulse width measurements. A total of 48 participants were assessed.

In our system, the pulse width was defined as the maximum pulse width during a pulse period, which was determined by counting the number of valid channels (those with maximum amplitudes ≥50% of the maximum channel). Figure 12 shows two typical cross-sectional curves of the arterial pulse pressure at different times from pulse onset to percussion peak P1. The x-axis represents the channel number and the y-axis represents the pressure. Cross-sectional curves were obtained by fitting the amplitudes at different channels. There were eight and three valid channels in Figure 12a,b, respectively. Figure 13 shows a summary of the pulse width measurements. A total of 48 participants were assessed.

**Figure 14:**
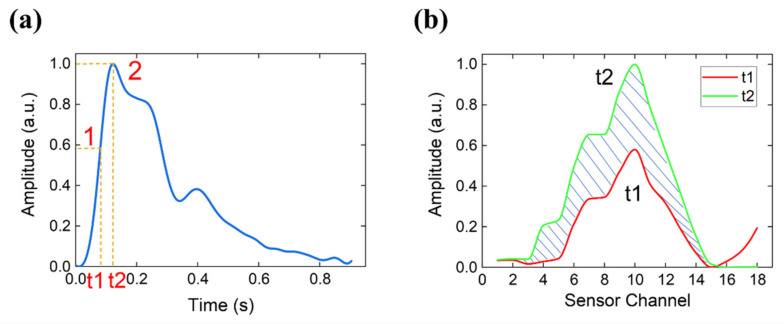
(**a**) Typical pulse waveform within a pulse period. (**b**) Curves formed by signals from all sensors sampled at different time points.

It is impossible to measure the minimum pulse width using a pressure-sensor array. Therefore, we recorded the pulse signals during a pulse period and used the cross-sectional curves at t1 and t2 to calculate the dynamic pulse width. The definitions of t1 and t2 are shown in Figure 14a. The parameter t2 is the time from pulse onset to the percussion peak P1, and t1=0.6×t2. To quantify the dynamic pulse width, we propose the indicator D_s_ as follows:

(1) $$D_{s}=\frac{\mathrm{Area}\;\mathrm{of}\;\mathrm{the}\;\mathrm{shadow}\;\mathrm{parts}}{\mathrm{Area}\;\mathrm{under}\;\mathrm{the}\;\mathrm{green}\;\mathrm{curve}}$$

where the area of the shadow is equal to the area under the green curve minus the area under the red curve, as shown in Figure 14b. Since the waveform amplitude does not affect the area indicator D_s_, the curve amplitudes are normalized to the range of 0–1 according to the maximum amplitude of the green curve.

**Figure 15:**
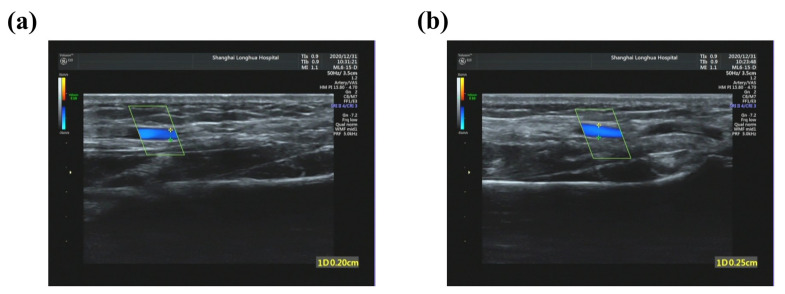
Diameter of Volunteer 1’s radial artery in diastole and systole was measured using an ultrasound instrument. (**a**) Diastolic diameter is 0.20 cm and (**b**) systolic diameter is 0.25 cm.

The pulses of the three volunteers were measured using the system, and their respective D_s_ values were calculated. Changes in radial artery diameters were also measured using an ultrasound instrument (GE LOGIQ 5 EXPERT, General Electric Company, Boston, MA, USA) at Shanghai Longhua Hospital. Figure 15 shows the ultrasound images of the radial artery in diastole and systole for volunteer 1. The difference between the systolic and diastolic values (∆D) for the participants were 0.05, 0.05, and 0.03 cm, which are consistent qualitatively with the calculated indicators of 0.331, 0.276, and 0.175. Detailed results are presented in Table 1.

### 3.6. PWV Acquisition

The PWV is the propagation velocity of pulse signals along blood vessels, which is an important parameter for characterizing human blood vessels and is the gold standard for detecting arterial stiffness [46].

To obtain the exact distances for PWV measurements, we measured the pulse signals at the brachial artery and the radial artery of the upper extremity using finger-worn sensor arrays. The PWV was calculated based on the distance between the two sensor arrays, L, and the pulse transit time (PTT).

(2) $$\mathrm{PWV}\;=\;\frac{L}{\mathrm{PTT}}$$

**Figure 16:**
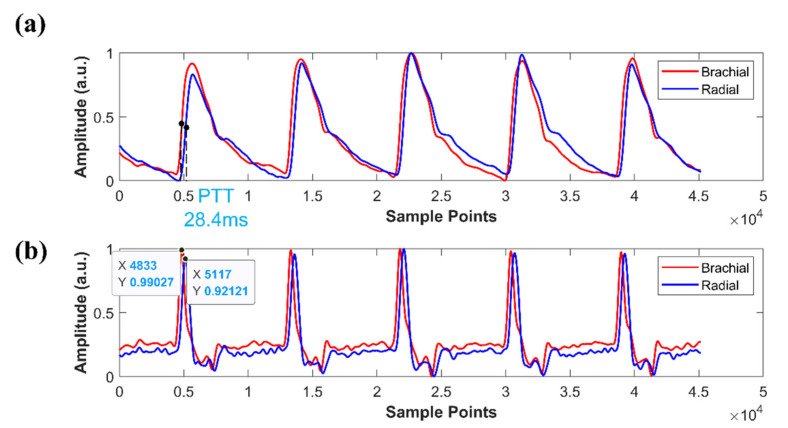
PTT calculation. (**a**) Brachial and radial artery pulse waveforms. (**b**) The first derivative pulse waveforms.

In this study, PTT was defined as the time lag between the maximum slope points of the brachial and radial artery pulse waveforms during the systolic period, as shown in Figure 16a. The maximum slope points are the maxima of the first derivative curve of the pulse signal, as shown in Figure 16b. Since the waveform amplitude does not affect the location of the maximum slope points, all signals are normalized to between 0 and 1.

**Figure 17:**
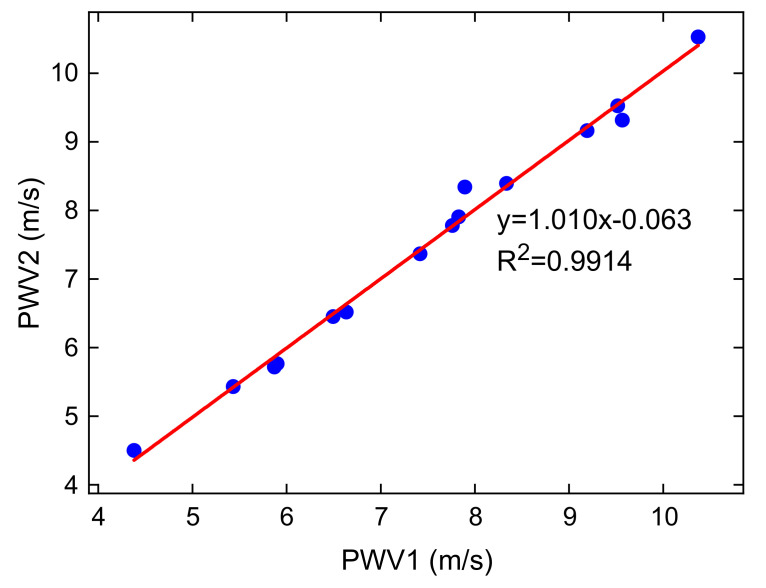
The fitting result of the two PWV measurements.

We measured the PWV of 15 subjects twice to confirm the reliability of PWV measurements. We used a tape measure to measure the distance from the brachial artery to the radial artery with an accuracy of 0.1 cm. The recording time for each person was 5s, and the subject was asked to sit in a chair calmly and not perform vigorous exercises for at least 30 min before the measurement was performed. Then, we used an algorithm in MATLAB 2019a to calculate the PWV value of each pulse period; the average PWV over all periods was taken as the final PWV value. The results of the two measurements are presented in Table 2. All PWV results are between 4.5 and 10.6 m/s, which is consistent with the PWV value of 5–10 m/s reported in the reported literature [46]. Furthermore, the repeatability was high between the two measurements and the difference between the two measurements was small. The average error between the two measurements was 0.11 m/s. Figure 17 shows the correlation between the two measurement trials.

As the proposed pulse acquisition system uses a small sensor array to measure pulse signals, there will be multiple sensors detecting the pulse signals at one location. Therefore, adjacent sensors have very similar signals, which makes the system more reliable than a single sensor.

### 3.7. Discussion

The proposed pulse acquisition system offers several unique advantages over previous designs. Table 3 lists the pulse measurement systems that have been previously reported for detecting wrist radial arterial pulses. The sensors used in this study are much smaller than those in the previous studies. We integrated 18 sensors to form a flexible pressure-sensor array with a sensitive length of 11.6 mm, which enables easy and accurate positioning of the sensor array on the radial artery and the measurements of pulse width and dynamic pulse width. The proposed flexible sensor array can be used on finger-worn applications, as well as wristbands and smartwatches for wearable monitoring. The pulse wave velocity can also be measured with the system, which may provide more information for pulse diagnosis.

## 4. Conclusions

This paper presents a radial artery pulse acquisition system based on finger-worn dense pressure-sensor arrays that were fabricated by packaging 18 ultra-small MEMS pressure sensors (0.4 mm × 0.4 mm × 0.2 mm each) on FPCs with a pitch of 0.65 mm. The pulse sensor arrays were calibrated using a custom-made system. Each sensor in the array was connected to an AD8221 instrumentation amplifier. A total of 54 instrumentation amplifiers were used for the system. The outputs of the instrumentation amplifiers were then sampled using a NI6255 data acquisition card.

TCM doctors can wear the pressure-sensor arrays on the fingers while palpating the pulses in order to record the pulse signal simultaneously. As the array has an effective span of 11.6 mm, which is 3–5 times the diameter of the human radial artery, quick and accurate positioning of the sensor array on the radial artery is achievable. Furthermore, high-quality pulse signals can be acquired under different pressures by well-trained doctors. As the pitches are much smaller than the diameter of the radial artery, the three-dimensional pulse envelope images, the width, and the dynamic width of the pulse signals can all be measured with the system. The pulse widths of 48 subjects were measured using the system, and the results indicated that the pulse widths could be effectively determined. The dynamic pulse widths of the three volunteers were also measured, and the results were qualitatively consistent with those obtained by color Doppler ultrasound. Finally, we demonstrated that the pulse wave velocity can also be obtained by measuring the phase transit time between the pulse signals at the brachial artery and the radial artery using the finger-worn sensor arrays. This new sensor array may offer a valuable pulse acquisition system for TCPD practitioners that overcomes many of the limitations of previous systems.

## Figures and Tables

**Table 1:** Dynamic pulse width results for three volunteers.

Volunteer	Diastolic Diameter (cm)	Systolic Diameter (cm)	ΔD (cm)	D_s_
1	0.20	0.25	0.05	0.331
2	0.21	0.26	0.05	0.276
3	0.19	0.22	0.03	0.175

**Table 2:** PWV values in two measurements.

Volunteer	L (cm)	PWV1 (m/s)	PWV2 (m/s)	abs (PWV1-PWV2) (m/s)
1	21.0	7.76	7.78	0.02
2	22.5	6.49	6.45	0.04
3	21.0	7.89	8.34	0.45
4	21.0	9.19	9.16	0.03
5	21.5	7.42	7.37	0.05
6	21.0	6.63	6.52	0.11
7	21.0	9.52	9.53	0.01
8	21.5	8.34	8.39	0.05
9	23.0	9.57	9.32	0.25
10	21.0	5.87	5.72	0.15
11	21.5	7.83	7.91	0.08
12	20.5	5.43	5.43	0.00
13	20.5	10.37	10.53	0.16
14	20.5	4.38	4.50	0.12
15	21.0	5.90	5.77	0.13
Mean				0.11
Min				0.00
Max				0.45

**Table 3:** Comparison of the proposed sensor array to other pulse measurement systems.

System	Tyan et al. [32]	Hu et al. [18]	Wang et al. [37]	Jin et al. [36]	Chen et al. [40]	Proposed
Number of sensors	One	3 × 4	Three main sensor 12 × 3 sub-sensors	Three	3 × 4	3 × 18
Single sensor size(mm)	Φ12	2.5 × 2.5	Main: 5 × 3 Sub: 0.8 × 8	5.6 × 5.6	5.5 × 3.6	0.4 × 0.4
Flexible sensor array	No	No	No	No	Yes	Yes
Wearable	No	No	No	No	No	Yes
Sensor positioning	No	No	Yes	No	No	Yes
Pulse width information	No	No	Yes	No	Yes	Yes
Pulse wave velocity measurement	No	No	No	No	No	Yes
Publication Year	2008	2012	2014	2019	2019	2021

## Data Availability

Data sharing not applicable.

## Supplementary Materials

The following are available online at https://www.mdpi.com/article/10.3390/mi12050569/s1, Figure S1: The 3D pulse envelope image, Figure S2: Single-point pulse waves.
