# Supplementary figures and images for: Three-Dimensional Arterial Pulse Signal Acquisition in Time Domain Using Flexible Pressure-Sensor Dense Arrays
Source: Micromachines (Basel). 2021 May 17;12(5):569. doi: 10.3390/mi12050569 (PMC8156466; doi:10.3390/mi12050569)

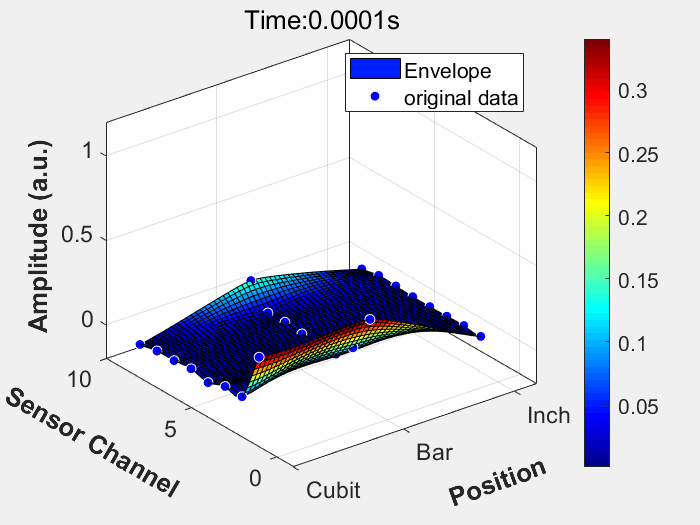

Supplement: Supplementary file 1 [file micromachines-12-00569-s001.zip › micromachines-1211255-supplementary/Figure_S1 The 3D pulse envelope image.gif]

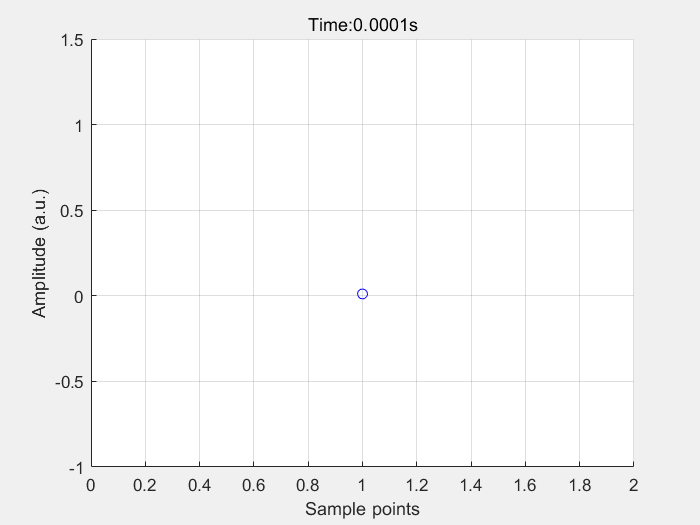

Supplement: Supplementary file 1 [file micromachines-12-00569-s001.zip › micromachines-1211255-supplementary/Figure_S2 Single-point pulse waves.gif]
